# A review of wearable sensors and systems with application in rehabilitation

**DOI:** 10.1186/1743-0003-9-21

**Published:** 2012-04-20

**Authors:** Shyamal Patel, Hyung Park, Paolo Bonato, Leighton Chan, Mary Rodgers

**Affiliations:** 1 Department of Physical Medicine and Rehabilitation, Harvard Medical School, Spaulding Rehabilitation Hospital, Boston, MA, USA; 2 Department of Electrical and Computer Engineering, Northeastern University, Boston, MA, USA; 3 Rehabilitation Medicine Department Clinical Center, National Institutes of Health, Bethesda, MD, USA; 4 Harvard-MIT Division of Health Sciences and Technology, Cambridge, MA, USA; 5 Department of Physical Therapy and Rehabilitation Science, University of Maryland School of Medicine, Baltimore, MD, USA; 6 National Institute of Biomedical Imaging and Bioengineering, National Institutes of Health, Bethesda, MD, USA

**Keywords:** Wearable sensors and systems, Home monitoring, Telemedicine, Smart home

## Abstract

The aim of this review paper is to summarize recent developments in the field of wearable sensors and systems that are relevant to the field of rehabilitation. The growing body of work focused on the application of wearable technology to monitor older adults and subjects with chronic conditions in the home and community settings justifies the emphasis of this review paper on summarizing clinical applications of wearable technology currently undergoing assessment rather than describing the development of new wearable sensors and systems. A short description of key enabling technologies (i.e. sensor technology, communication technology, and data analysis techniques) that have allowed researchers to implement wearable systems is followed by a detailed description of major areas of application of wearable technology. Applications described in this review paper include those that focus on health and wellness, safety, home rehabilitation, assessment of treatment efficacy, and early detection of disorders. The integration of wearable and ambient sensors is discussed in the context of achieving home monitoring of older adults and subjects with chronic conditions. Future work required to advance the field toward clinical deployment of wearable sensors and systems is discussed.

## Introduction

The US health care system faces daunting challenges. With the improvements in health care in the last few decades, residents of industrialized countries are now living longer, but with multiple, often complex, health conditions [1-3]. Survival from acute trauma has also improved, but this is associated with an increase in the number of individuals with severe disabilities [4]. From an epidemiological standpoint, the cohort of "baby boomers" in the US is now reaching an age at which they will begin to severely stress the Medicare system. Finally, recent health care reform efforts may add 32 million newly insured patients to the health care system in the next few years [5].

These altered demographics raise some fundamental questions

• How do we care for an increasing number of individuals with complex medical conditions?

• How do we provide quality care to those in areas with reduced access to providers?

• How do we maximize the independence and participation of an increasing number of individuals with disabilities?

Cleary, answers to these questions will be complex and will require changes into how we organize and pay for health care. However, part of the solution may lie in how and to what extent we take advantage of recent advances in information technology and related fields. Currently, there exist technologies that hold great promise to expand the capabilities of the health care system, extending its range into the community, improving diagnostics and monitoring, and maximizing the independence and participation of individuals. This paper will discuss these technologies in depth, with a focus on remote monitoring systems based on wearable technology. We chose to focus on these technologies because recent developments in wearable sensor systems have led to a number of exciting clinical applications.

Wearable sensors have diagnostic, as well as monitoring applications. Their current capabilities include physiological and biochemical sensing, as well as motion sensing [6,7]. It is hard to overstate the magnitude of the problems that these technologies might help solve. Physiological monitoring could help in both diagnosis and ongoing treatment of a vast number of individuals with neurological, cardiovascular and pulmonary diseases such as seizures, hypertension, dysrthymias, and asthma. Home based motion sensing might assist in falls prevention and help maximize an individual's independence and community participation.

Remote monitoring systems have the potential to mitigate problematic patient access issues. Nearly 20% of those in the US live in rural areas, but only 9% of physicians work in rural areas [8]. Access may get worse over time as many organizations are predicting a shortfall in primary care providers as health care reform provides insurance to millions of new patients [9]. There is a large body of literature that describes the disparities in care faced by rural residents [8]. Compared to those in urban areas, those in rural areas travel 2 to 3 times farther to see a physician, see fewer specialists, and have worse outcomes for such common conditions as diabetes, and heart attack [9,10]. Wearable sensors and remote monitoring systems have the potential to extend the reach of specialists in urban areas to rural areas and decrease these disparities.

**Figure 1:**
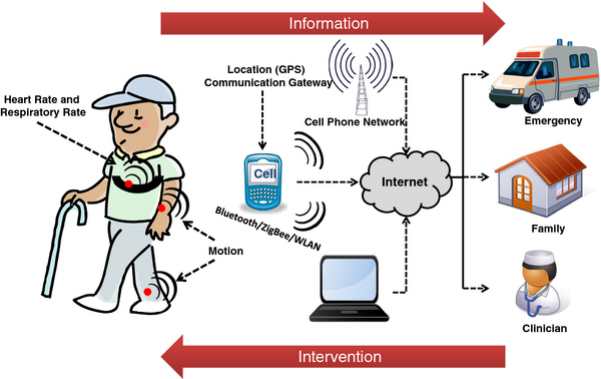
**Illustration of a remote health monitoring system based on wearable sensors**. Health related information is gathered via body-worn wireless sensors and transmitted to the caregiver via an information gateway such as a mobile phone. Caregivers can use this information to implement interventions as needed.

A conceptual representation of a system for remote monitoring is shown in Figure 1. Wearable sensors are used to gather physiological and movement data thus enabling patient's status monitoring. Sensors are deployed according to the clinical application of interest. Sensors to monitor vital signs (e.g. heart rate and respiratory rate) would be deployed, for instance, when monitoring patients with congestive heart failure or patients with chronic obstructive pulmonary disease undergoing clinical intervention. Sensors for movement data capturing would be deployed, for instance, in applications such as monitoring the effectiveness of home-based rehabilitation interventions in stroke survivors or the use of mobility assistive devices in older adults. Wireless communication is relied upon to transmit patient's data to a mobile phone or an access point and relay the information to a remote center via the Internet. Emergency situations (e.g. falls) are detected via data processing implemented throughout the system and an alarm message is sent to an emergency service center to provide immediate assistance to patients. Family members and caregivers are alerted in case of an emergency but could also be notified in other situations when the patient requires assistance with, for instance, taking his/her medications. Clinical personnel can remotely monitor patient's status and be alerted in case a medical decision has to be made.

Despite the potential advantages of a remote monitoring system relying on wearable sensors like the one described above, there are significant challenges ahead before such a system can be utilized on a large scale. These challenges include technological barriers such as limitations of currently available battery technology as well cultural barriers such as the association of a stigma with the use of medical devices for home-based clinical monitoring. In the following section, we discuss key technologies enabling the development and deployment of wearable technologies and remote monitoring systems. The next section describes wearable and ambient sensor technologies that are essential components of systems to monitor patients in the home and community settings. Examples of applications of these technologies largely taken from a National Science Foundation initiated study of European projects focused on rehabilitation technology [11] are then presented. Conclusions and future developments that we foresee in the field of remote monitoring of patients' status via wearable technology are discussed in the final section.

## Key enabling technologies

Wearable systems for patients' remote monitoring consist of three main building blocks: 1) the sensing and data collection hardware to collect physiological and movement data, 2) the communication hardware and software to relay data to a remote center, and 3) the data analysis techniques to extract clinically-relevant information from physiological and movement data. Recent advances in sensor technology, microelectronics, telecommunication, and data analysis techniques have enabled the development and deployment of wearable systems for patients' remote monitoring. Researchers have relied upon advances in the above-mentioned fields to address shortcomings of ambulatory technologies (e.g. Holter monitors) that had previously prevented long-term monitoring of patients' status in the home and community settings.

**Figure 2:**
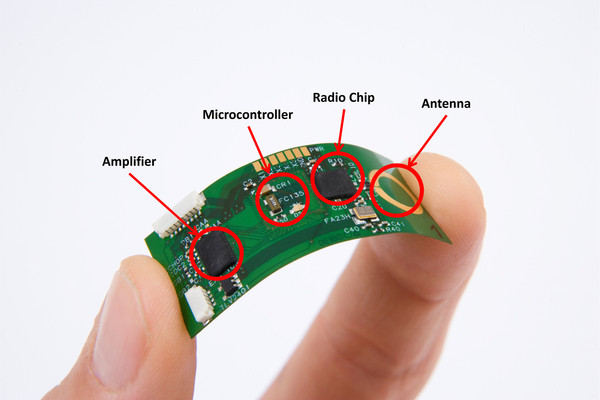
**Flexible wireless ECG sensor with a fully functional microcontroller by IMEC**. Developments in the field of flexible electronics are expected to lead to the advent of smaller, lighter and more comfortable wearable systems. (Courtesy of IMEC, The Netherlands).

The miniaturization of sensors and electronic circuits based on the use of microelectronics has played a key role in the development of wearable systems. One of the major hurdles to the adoption of sensing technology, especially for wearable applications, has been the size of the sensors and front-end electronics that, in the past, made the hardware to gather physiological and movement data too obtrusive to be suitable for long-term monitoring applications. Recent developments in the field of microelectronics have allowed researchers to develop miniature circuits entailing sensing capability, front-end amplification, microcontroller functions, and radio transmission. The flexible circuit shown in Figure 2 is an example of such technology and allows one to gather physiological data as well as transmit the data wirelessly to a data logger using a low-power radio. Particularly relevant to applications in the field of rehabilitation are advances in technology to manufacture microelectromechanical systems (MEMS). MEMS technology has enabled the development of miniaturized inertial sensors that have been used in motor activity and other health status monitoring systems. By using batch fabrication techniques, significant reduction in the size and cost of sensors has been achieved. Microelectronics has also been relied upon to integrate other components, such as microprocessors and radio communication circuits, into a single integrated circuit thus resulting in System-on-Chip implementations [12].

**Figure 3:**
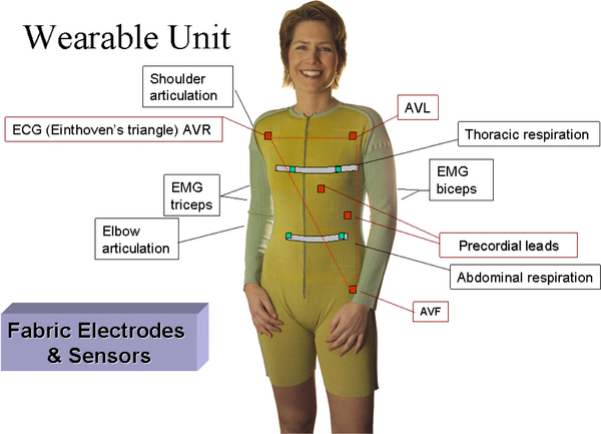
**Example of e-textile system for remote, continuous monitoring of physiological and movement data**. Embedded sensors provide one with the capability of recording electrocardiographic data (ECG) using different electrode configurations as well as electromyographic (EMG) data. Additional sensors allow one to record thoracic and abdominal signals associated with respiration and movement data related to stretching of the garment with shoulder movements. (Courtesy of Smartex, Italy).

Advances in material science have enabled the development of e-textile based systems. These are systems that integrate sensing capability into garments. The example shown in Figure 3 demonstrates how sensors can be embedded in a garment to collect, for instance, electrocardiographic and electromyographic data by weaving electrodes into the fabric and to gather movement data by printing conductive elastomer-based components on the fabric and then sensing changes in their resistance associated with stretching of the garment due to subject's movements. Rapid advances in this field promise to deliver technology that will soon allow one to print a full circuit board on fabric.

Health monitoring applications of wearable systems most often employ multiple sensors that are typically integrated into a sensor network either limited to body-worn sensors or integrating body-worn sensors and ambient sensors. In the early days of body-worn sensor networks (often referred to as "body sensor networks"), the integration of wearable sensors was achieved by running "wires" in pockets created in garments for this purpose to connect body-worn sensors. An example of this technology is the MIThril system [13]. Such systems by design were not suitable for long-term health monitoring. Recently developed wearable systems integrate individual sensors into the sensor network by relying on modern wireless communication technology. During the last decade, we have witnessed tremendous progress in this field and the development of numerous communication standards for low-power wireless communication. These standards have been developed keeping in mind three main requirements: 1) low cost, 2) small size of the transmitters and receivers, and 3) low power consumption. With the development of IEEE 802.15.4/ZigBee [14] and Bluetooth, tethered systems have become obsolete. The recently developed IEEE 802.15.4a standard based on Ultra-wide-band (UWB) impulse radio opens the door for low-power, low-cost but high data rate sensor network applications with the possibility of highly accurate location estimation [15].

Most monitoring applications require that data gathered using sensor networks be transmitted to a remote site such as a hospital server for clinical analysis. This can be achieved by transmitting data from the sensor network to an information gateway such as a mobile phone or personal computer. By now most developed countries have achieved almost universal broadband connectivity. For in-home monitoring, sensor data can be aggregated using a personal computer and transmitted to the remote site over the Internet. Also, the availability of mobile telecommunication standards such as 4 G means that pervasive continuous health monitoring is possible when the patient is outside the home environment.

**Figure 4:**
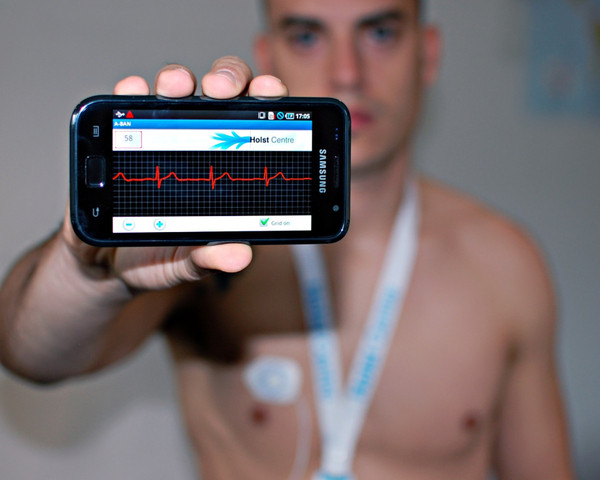
**Smart phone based ECG monitoring system by IMEC**. The Android based mobile application allows low power ECG sensors to communicate wirelessly with the phone. With increasing computational and storage capacity and ubiquitous connectivity, smart phones are expected to truly enable continuous health monitoring. (Courtesy of IMEC, The Netherlands).

Mobile phone technology has had a major impact on the development of remote monitoring systems based on wearable sensors. Monitoring applications relying on mobile phones such as the one shown in Figure 4 are becoming commonplace. Smart phones are broadly available. The global smart phone market is growing at an annual rate of 35% with an estimated 220 million units shipped in 2010 [16]. Smart phones are preferable to traditional data loggers because they provide a virtually "ready to use" platform to log data as well as to transmit data to a remote site. Besides being used as information gateways, mobile devices can also function as information processing units. The availability of significant computing power [17] in pocket-sized devices makes it possible to envision ubiquitous health monitoring and intervention applications.

In addition, most mobile devices now include an integrated GPS tracking system thus making it possible to locate patients in case of an emergency. Also, as storage and computation becomes more and more cloud based, health monitoring systems can become low-cost, platform-independent, rapidly deployable and universally accessible [18,19]. Monitoring devices can become simpler and cheaper as the computation is pushed to the cloud. This enables users to buy off-the-shelf devices and access customized monitoring applications via cloud-based services [20]. Cloud-based systems can prove especially useful for bringing health care services to rural areas [21]. In addition, monitoring applications deployed via the cloud can be easily updated without requiring that the patient installs any software on his/her personal monitoring device, thus making system maintenance quick and cost effective.

Finally, the massive amount of data that one can gather using wearable systems for patient's status monitoring has to be managed and processed to derive clinically-relevant information. Data analysis techniques such as signal processing, pattern recognition, data mining and other artificial intelligence-based methodologies have enabled remote monitoring applications that would have been otherwise impossible. Although a discussion of the various techniques used to process and analyze wearable sensor data is outside the scope of this review paper, one cannot emphasize enough the fact that data processing and analysis techniques are an integral part of the design and development of remote monitoring systems based on wearable technology.

## Sensing technology

**Figure 5:**
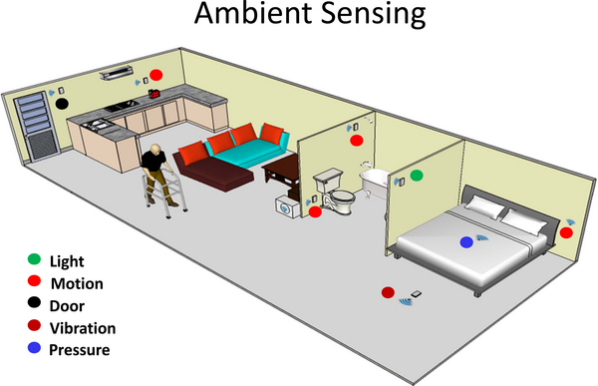
**Ambient sensors can unobtrusively monitor individuals in the home environment**. Ambient sensors can monitor activity patterns, sleep quality, bathroom visits etc. and provide alerts to caregivers when abnormal patterns are observed. Such sensors are expected to make the home of the future smarter and safer for patients living with chronic conditions.

In this section, we provide information concerning the sensors used in remote monitoring systems. Information gathered using body-worn (i.e. wearable) sensors is collected ubiquitously thanks to the technologies mentioned in the previous section of this review paper. Wearable sensors are often combined with ambient sensors when subjects are monitored in the home environment as schematically shown in Figure 5. The combination of wearable and ambient sensors is of great interest in several applications in the field of rehabilitation. For instance, when monitoring older adults while deploying interventions to improve balance control and reduce falls, one would be interested in using wearable sensors to track motion and vital signs. Specifically-designed data analysis procedures would then be used to detect falls via processing of motion and vital sign data. In this context, ambient sensors could be used in conjunction with wearable sensors to improve the accuracy of falls detection and, most importantly, to enable the detection of falls even at times when subjects do not wear the sensors. This section provides a summary of the state of the art in wearable sensor technology and the development of ambient sensors.

### Wearable sensors

Physiological measures of interest in rehabilitation include heart rate, respiratory rate, blood pressure, blood oxygen saturation, and muscle activity. Parameters extracted from such measures can provide indicators of health status and have tremendous diagnostic value. Until recently, continuous monitoring of physiological parameters was possible only in the hospital setting. But today, with developments in the field of wearable technology, the possibility of accurate, continuous, real-time monitoring of physiological signals is a reality.

Integrating physiological monitoring in a wearable system often requires ingenious designs and novel sensor locations. For example, Asada et al. [22] presented a ring sensor design for measuring blood oxygen saturation (SpO_2_) and heart rate. The ring sensor was completely self-contained. Worn on the base of the finger (like a ring), it integrated techniques for motion artifact reduction, which were designed to improve measurement accuracy. Applications of the ring sensor ranged from the diagnosis of hypertension to the management of congestive heart failure. A self-contained wearable cuff-less photoplethysmographic (PPG) based blood pressure monitor was subsequently developed by the same research group [23]. The sensor integrated a novel height sensor based on two MEMS accelerometers for measuring the hydrostatic pressure offset of the PPG sensor relative to the heart. The mean arterial blood pressure was derived from the PPG sensor output amplitude by taking into account the height of the sensor relative to the heart.

Another example of ingenious design is the system developed by Corbishley et al. [24] to measure respiratory rate using a miniaturized wearable acoustic sensor (i.e. microphone). The microphone was placed on the neck to record acoustic signals associated with breathing, which were band-pass filtered to obtain the signal modulation envelope. By developing techniques to filter out environmental noise and other artifacts, the authors managed to achieve accuracy greater than 90% in the measurement of breathing rate. The authors also presented an algorithm for the detection of apneas based on the above-described sensing technology.

In recent years, physiological monitoring has benefited significantly from developments in the field of flexible circuits and the integration of sensing technology into wearable items [25]. An ear-worn, flexible, low-power PPG sensor for heart rate monitoring was introduced by Patterson et al. [26]. The sensor is suited for long-term monitoring due to its location and unobtrusive design. Although systems of this type have shown promising results, additional work appears to be necessary to achieve motion artifact reduction [27,28]. Proper attenuation of motion artifacts is essential to the deployment of wearable sensors. Some of the problems due to motion artifacts could be minimized by integrating sensors into tight fitting garments. A comparative analysis of different wearable systems for monitoring respiratory function was presented by Lanata et al. [29]. The analysis showed that piezoelectric pneumography performs better than spirometry. Nonetheless, further advances in signal processing techniques to mitigate motion artifacts are needed.

Biochemical sensors have recently gained a great deal of interest among researchers in the field of wearable technology. These types of sensors can be used to monitor the bio-chemistry as well as levels of chemical compounds in the atmosphere (e.g. to facilitate monitoring people working in hazardous environments). From a design point of view, biochemical sensors are perhaps the most complex as they often require collection, analysis and disposal of body fluids. Advances in the field of wearable biochemical sensors has been slow, but research has recently picked up pace due to the development of micro and nano fabrication technologies [12]. For example, Dudde et al. [30] developed a minimally-invasive wearable closed-loop quasi-continuous drug infusion system that measures blood glucose levels and infuses insulin automatically. The glucose monitor consists of a novel silicon sensor that continuously measures glucose levels using a microperfusion technique and continuous infusion of insulin is achieved by a modified advanced insulin pump. The device has integrated Bluetooth communication capability for displaying and logging data and receiving commands from a personal digital assistant (PDA) device.

An array of bio-chemical sensors has been developed as part of the BIOTEX project, supported by the European Commission. Specifically, the BIOTEX project deals with the integration of bio-chemical sensors into textiles for monitoring body fluids. Within this project, researchers have developed a textile based fluid collecting system and sensors for in-vitro and in-vivo testing of pH, sodium and conductivity from body sweat [31,32]. By in-vitro and in-vivo testing of the wearable system, researchers have shown that the system can be used for real-time analysis of sweat during physical activity. As part of a similar project called ProeTEX, Curone et al. [33] developed a wearable sensorized garment for firefighters, which integrates a CO_2 _sensor with sensors to measure movement, environmental and body temperature, position, blood oxygen saturation, heart rate and respiration rate. The ProeTEX system can warn the firefighters of a potentially dangerous environment and also provide information about their well being to the control center. The systems developed in the above-mentioned projects could be relied upon to design robust e-textile based wearable systems for remote health monitoring applications.

There has been a growing interest in the development of self contained lab-on-a-chip systems. Such systems can revolutionize point-of-care medical testing and diagnosis by making testing and diagnosis fast, cheap and easily accessible. Wang et al. [34] developed a system-on-chip (SOC), which integrates a pH and temperature sensor, for remote monitoring applications. Their SOC includes generic sensor interface, ADC, microcontroller, a data encoder and a frequency-shift keying RF transmitter. Similarly, Ahn et al. [35] developed a low-cost disposable plastic lab-on-a-chip device for biochemical detection of parameters such as blood gas concentration and glucose. The biochip contains an integrated biosensor array for detecting multiple parameters and uses a passive microfluidic manipulation system instead of active microfluidic pumps.

Finally, applications in rehabilitation of remote monitoring systems relying on wearable sensors [36] have largely relied upon inertial sensors for movement detection and tracking. Inertial sensors include accelerometers and gyroscopes. Often, magnetometers are used in conjunction with them to improve motion tracking. Today, movement sensors are inexpensive, small and require very little power, making them highly attractive for patient monitoring applications.

### Ambient sensors

Examples of instrumented environments include sensors and motion detectors on doors that detect opening of, for instance, a medicine cabinet, refrigerator, or the home front door [37]. This approach has the characteristic of being totally unobtrusive and of avoiding the problem of misplacing or damaging wearable devices. "Smart home" technology that includes ambient and environmental sensors has been incorporated in a variety of rehabilitation related applications. One such application is ambient assisted living (AAL) that refers to intelligent systems of health assistance in the individual's living environment [38]. It covers concepts, products and services that interlink and improve new technologies and the social environment. AAL technologies are embedded (distributed throughout the environment or directly integrated into appliances or furniture), personalized (tailored to the users' needs), adaptive (responsive to the user and the user's environment) and anticipatory (anticipating users' desires as far as possible without conscious mediation). Stefanov et al provide a summary of the various types of devices that can be installed in smart homes, and the associated target user populations [39].

Remote monitoring of patient status and self-management of chronic conditions represent the most often pursued applications of AAL technologies. The combination of wearable and ambient sensors is being explored and prototypes are being developed. A relevant application in the field of rehabilitation relates to the identification of a patient's patterns of activity and on providing suggestions concerning specific behaviors and exercises for self-management of health conditions. In this context, information gathered using wearable sensors is augmented by information gathered using ambient sensors. Data collected using, for instance, body-worn accelerometers could be augmented by motion sensors distributed throughout the home environment to determine the type and intensity of the activities performed by an individual. Accordingly, an individual undergoing monitoring who suffers from, for instance, chronic obtrusive pulmonary disease could receive feedback about not overexerting himself/herself and the performance of rehabilitation exercises that would be prescribed in order to maintain a satisfactory functional level.

Innovative solutions for recognizing emergencies in the home can be achieved through a combination of monitoring vital parameters of the person living at home as well as supervising the conditions of domestic appliances [40]. Personal safety can be improved if vital data measures are combined with the monitoring and control of devices in the household. Remote monitoring of potential sources of danger increases the individual sense of security and can make life much easier and more comfortable (e. g. checking whether the stove or the coffee machine has been switched off and to be able to turn them off remotely if necessary). Sensors embedded in electrical devices and in doors and windows may be integrated into an easy-to-use house-control system that also provides improved personal safety and security [41]. An intelligent system may issue a reminder to switch off devices and/or lights in an apartment or not to forget the pill box or the mobile terminal needed to inform friends or neighbors when necessary.

Several smart home projects are currently ongoing including the Technology Research for Independent Living (TRIL) Center in Ireland [42], the TigerPlace [43] in Missouri, the Oregon Center for Aging and Technology (ORCATECH) [44] in Oregon, the University of Rochester Center for Future Health [45], The University of Florida Gator-Tech Smart House [46], the Georgia Institute of Technology Aware Home [47], and the Massachusetts Institute of Technology PlaceLab [48]. The main aim of such projects is to explore the use of ambient and/or wearable sensing technology to monitor the well-being of individuals in the home environment.

## Applications

This section provides about a review of applications of wearable and ambient sensors and systems that are relevant to the field of rehabilitation. The material is organized in five sub-sections devoted to summarizing applications focused on: 1) health and wellness monitoring, 2) safety monitoring, 3) home rehabilitation, 4) assessment of treatment efficacy, and 5) early detection of disorders.

### Health & wellness monitoring

As the world population is aging and health care costs are increasing, several countries are promoting "aging in place" programs which allow older adults and individuals with chronic conditions to remain in the home environment while they are remotely monitored for safety and for the purpose of facilitating the implementation of clinical interventions.

Monitoring activities performed by older adults and individuals with chronic conditions participating in "aging in place" programs has been considered a matter of paramount importance. Accordingly, extensive research efforts have been made to assess the accuracy of wearable sensors in classifying activities of daily living (ADL). Mathie et al [49] showed the feasibility of using accelerometers to identify the performance of ADL by older adults monitored in the home environment. Sazonov et al [50] developed an in-shoe pressure and acceleration sensor system that was used to classify activities including sitting, standing, and walking with the ability of detecting whether subjects were simultaneously performing arm reaching movements. Giansanti et al [51] developed an accelerometer-based device designed for step counting in patients with Parkinson's disease. Aziz et al [52] used wearable sensors to monitor the recovery of patients after abdominal surgery. Several research projects have suggested that activity monitoring for wellness applications has great potential to increase exercise compliance in populations at risk. For example, wearable technology has been used to monitor physical activities in obese individuals and to facilitate the implementation of clinical interventions based on encouraging an active and healthy lifestyle [53-56].

Long-term monitoring of physiological data can lead to improvements in the diagnosis and treatment, for instance, of cardiovascular diseases. Commercially available technology provides one with the ability to achieve long-term monitoring of heart rate, blood pressure, oxygen saturation, respiratory rate, body temperature and galvanic skin response. Clinical studies are currently carried out to evaluate and validate the performance of wearable sensor platforms to monitor physiological data over long periods of time and improve the clinical management of patients, for instance, with congestive heart failure [57,58].

Several ongoing studies are focused on clinically assessing wearable systems developed as part of major research projects. For instance, LiveNet, a system developed at the MIT Media Laboratory that measures 3-D acceleration, ECG, EMG, and galvanic skin conductance, is under evaluation for monitoring Parkinsonian symptoms and detecting epileptic seizures [59]. LifeGuard is a custom data logger designed to monitor health status of individuals in extreme environments (space and terrestrial) [60]. The system has undergone testing in hostile environments with good results. As part of the FP5 program of the European Commission, a project named AMON resulted in the development of a wrist-worn device capable of monitoring blood pressure, skin temperature, blood oxygen saturation, and ECG. The device was developed to monitor high risk patients with cardio-respiratory problems [61]. Other projects worth mentioning that have been carried out as part of different programs of the European Commission are: MyHeart [62], WEALTHY [63,64], and MagIC [65,66]. These projects led to the development of garment-based wearable sensors aiming at general health monitoring of people in the home and community settings.

### Safety monitoring

A number of devices have been developed for safety monitoring applications, such as detecting falls and relaying alarm messages to a caregiver or an emergency response team. The Life Alert Classic by Life Alert Emergency Response Inc [67] and the AlertOne medical alert system [68] are examples of commercially-available devices designed for safety monitoring. These devices are simple emergency response devices consisting of a pendant or watch with a push button. Pressing the button, one has the ability to wirelessly relaying an alarm message to operators located in a remote call center. Other systems integrate sensors into the body-worn unit. For instance, the Wellcore system [69] employs advanced microprocessors and accelerometers to monitor the body's position. The system detects falls as distinct events from normal movements, and automatically relays a message to the designated response center or nurse call station. Another device in this category is the MyHalo™ by Halo Monitoring™. The system is worn as a chest strap and detects falls, while it monitors heart rate, skin temperature, sleep/wake patterns, and activity levels [70]. The BrickHouse system [71] equipped with an automatic fall detector and a manual panic button. Finally, among the numerous commercially-available systems, it is worth mentioning the ITTM EasyWorls [72], a system based on a mobile phone that is equipped with balance sensors which trigger automatic dialing SOS numbers if the system detects a sudden impact.

Reliable detection of falls via wearable sensors has been achieved by many research groups. Researchers at CSEM [73] developed an automatic fall detection system in the form of a wrist watch. The device implements functionalities such as wireless communication, automatic fall detection, manual alarm triggering, data storage, and a simple user interface. Even though the wrist is a challenging sensor location to detect a fall event, researchers on the project achieved 90% sensitivity and 97% specificity in the detection of simulated falls. Bourke et al [74] took an alternative approach and used a tri-axial accelerometer embedded in a custom-designed vest to detect falls. Bianchi et al [75] used instead a barometric pressure sensor as a surrogate measure of altitude to discriminate real fall events from normal activities of daily living. When tested in a cohort of 20 young healthy volunteers, the proposed method demonstrated considerable improvements in sensitivity and specificity compared to an existing accelerometer-based technique. Finally, among the numerous systems developed by researchers to detect falls, it is worth mentioning that Lanz et al [76] developed SmartFall, a system that relies on an accelerometer embedded in a cane to detect falls. The authors argued that canes are assistive devices that people widely use to overcome problems associated with balance disorders and therefore that embedding the system in the cane is a very appealing solution to achieve unobtrusive monitoring while assuring safety of individuals.

Recent advances in smart phone technology have led to their use in fall detection systems. Often, these systems combine fall detection with localization of the person who fell via a GPS-based method [77,78]. Yavuz et al [79] developed a fall detection system that relied upon the accelerometers available in smart phones and incorporated different algorithms for robust detection of falls. Their implementation leveraged the characteristics of the Android 2 operating system. The authors developed advanced signal processing techniques to achieve high accuracy of falls detection. Besides, the system provided subject's location using Google Maps. Using this approach, a warning about the fall and the location of the subject undergoing monitoring is transmitted to a caregiver or family member via SMS, email and Twitter messages. Ongoing research is geared toward the prevention of fall-related injuries. Numerous systems have been developed by leveraging airbag technology [80-83]. These systems rely upon wearable accelerometers and gyroscopes to trigger the inflation of the airbag when a fall is detected. Although these systems can potentially help to prevent fall-related injuries, further development is needed to miniaturize the airbag system that provides protection to the subject before an impact occurs.

Individuals with movement impairments require more specific approaches to detect or prevent falls. Bachlin et al [84] developed a system to detect freezing of gait (FOG), a commonly found gait symptom in Parkinson's disease that is highly related to falls. The system was designed to provide subjects with a rhythmic auditory signal aimed to stimulate the patient to resume walking when a FOG episode is detected. Smith and Bagley [85] developed a system to be used in children with difficulty in walking, which is known to be associated with frequent falls. They collected tri-axial accelerometer data and digital video recordings for over 50 hours from 35 children with cerebral palsy and 51 control subjects. The dataset was used to develop algorithms for automatic real-time processing of the accelerometer signals to monitor a child's level of activity and to detect falls [85]. Sposaro et al [86] focused their attention on older adults with dementia. These subjects require frequent caregivers' assistance to accomplish standard activities of daily living. The authors relied upon an Android application, iWander, which uses GPS and communication functions available via the smart phone, to provide tracking of subjects' location and assistance when needed. The system was shown to improve functional independence among dementia patients while decreasing the stress put on caregivers.

Another application of wearable sensors and systems that has received a great deal of attention among researchers and clinicians is the detection of epileptic seizures. Primary and secondary compulsive epileptic crises (EC) cause a sudden loss of consciousness. These events are accompanied by stereotypical movements that one can observe in association with characteristic changes in the electroencephalogram (EEG). During the acute phase, the subject is completely unable to interact with the environment. To detect EC, systems and methods relying upon wearable sensors have been proposed and evaluated. Electroencepholographic (EEG) sensors [87], 3D accelerometers on a wrist [88], combination of EMG and accelerometers [89], and electrodermal activity (EDA) [90] have been used to develop methods to distinguish EC from normal motor activities. Dalton et al [91] used a Nokia N810 and the SHIMMER platform of wearable sensors to detect seizure events.

An interesting recent development is the integration of various sensors and systems in a network for comprehensive safety monitoring and smart home health care applications. AlarmNet is an example of such systems. It collects and analyzes various data streams to monitor a resident's overall wellness, known medical conditions, activities of daily living, and emergency situations. The whole project deals not only with wearable sensing technology but also with security/privacy issues in patient's data transfer, and real-time data streaming [92]. A major contribution toward the development of new solutions in the field of wearable and ambient sensors and their integration in comprehensive safety monitoring and smart home health care applications is provided by the European AALIANCE (Ambient Assisted Living Innovation Alliance). AALIANCE is an active project that includes many research institutes, companies, and universities in Europe. The project aim is to define the necessary future R&D steps toward developing Ambient Assisted Living (AAL) solutions. The project builds infrastructure for practical applications of wearable technologies such as telemonitoring of patient's status and self-management of chronic diseases.

**Figure 6:**
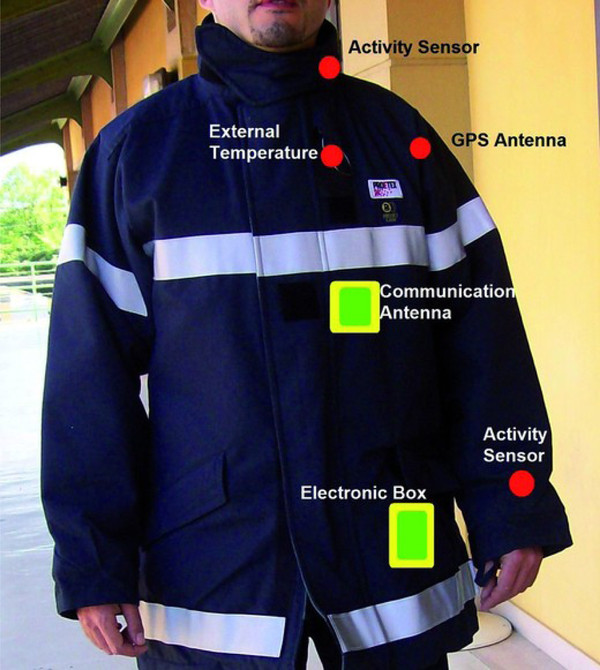
**The ProeTEX project aims to develop smart garments for emergency responders**. These smart garments integrate sensors, communication, processing and power management directly into the garment to continuously monitor emergency responders. (Courtesy of Smartex, Italy).

Safety monitoring applications typically require detection of emergency events. The sensing technology used for such applications must be extremely robust and reliable. A great deal work has been done toward developing wearable systems to monitor individuals working in hostile environments in response to emergency situations. The Proe-TEX project, carried out as part of the FP6 program of the European Commission, is an example of such work. The project resulted into the development of a new generation of smart garments to monitor emergency-disaster personnel (see Figure 6). These garments enable the detection of health status parameters of the users and environmental variables such as external temperature, presence of toxic gases, and heat flux passing through the garments. Extensive testing of the garments is being carried out both in laboratories, specialized in physiological measures, and in simulated fire-fighting scenarios [33,93]. Advances achieved in the above-mentioned projects could be used to design robust systems for home health monitoring to be deployed to detect emergency events such as falls and seizures.

### Home rehabilitation

An emerging area of application of wearable technology is the use of wearable sensors to facilitate the implementation of home-based rehabilitation interventions. Systems that aim to facilitate the implementation of rehabilitation exercise programs often leverage the combination of sensing technology and interactive gaming or virtual reality (VR) environments. For example, The Rehabilitation Engineering Research Center at the University of Southern California [94] is building on VR gaming to address compliance and motivation challenges [95]. VR simulation technology using specialized interface devices has been applied to improve motor skills in subjects undergoing rehabilitation to address functional deficits including reaching, hand function and walking. It has been proposed that such VR-based activities could be delivered in the home via a telerehabilitation approach to support patients' increased access to rehabilitation and preventive exercise programming. When this is put in an interactive game-based context, the potential exists to enhance the engagement and motivation needed to drive neuroplastic changes that underlie motor process maintenance and improvement. However, home-based systems need to be affordable and easy to deploy and maintain, while still providing the interactional fidelity required to produce the meaningful motor activity required to foster rehabilitative aims and promote transfer to real world activities.

**Figure 7:**
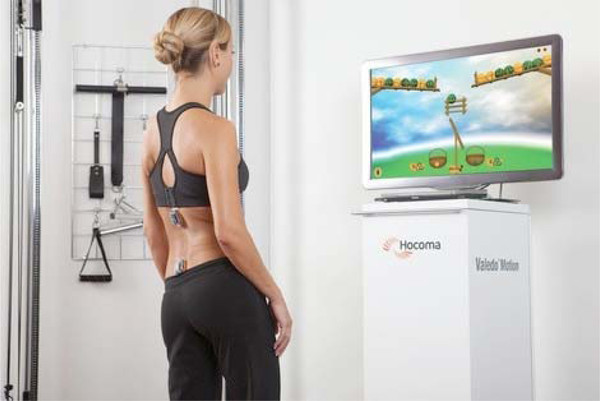
**The Valedo low back pain therapy system by Hocoma AG combines wireless wearable motion sensors with interactive games to provide an engaging way to perform therapeutic exercises**. Patients can set therapy goals, receive feedback on their performance and keep track of their progress. (Courtesy of Hocoma, Switzerland).

An example of such systems is the Valedo system by Hocoma AG shown in Figure 7. The Valedo system is a medical back training device, which improves patient's compliance and allows one to achieve increased motivation by real time Augmented Feedback based on trunk movements. It transfers trunk movements from two wireless sensors into a motivating game environment and guides the patient through exercises specifically designed for low back pain therapy. To facilitate challenging the patient and achieving efficient training, the exercises can be adjusted according to the patient's specific needs. Several other systems are currently under development. For instance, GE Healthcare is developing a wireless medical monitoring system that is expected to allow one to gather physiological and movement data thus facilitating rehabilitation interventions in the home setting. Another example of home-based rehabilitation technology is the Stroke Rehabilitation Exerciser developed by Philips Research [96]. The Stroke Rehab Exerciser coaches the patient through a sequence of exercises for motor retraining, which are prescribed by the physiotherapist and uploaded to a patient unit. A wireless inertial sensor system records the patient's movements, analyzes the data for deviations from a personal movement target and provides feedback to the patient and the therapist.

Major efforts have been made by European groups to develop systems suitable for home-based interventions that rely on wearable technology. A project that was part of the myHeart initiative [97,98] led to the development of a sensorized garment-based system to facilitate rehabilitation interventions in the home setting. The system allows patients to increase the amount of motor exercise they can perform independently, providing them with a real-time feedback based on wearable sensors embedded in the garment across the upper limb and trunk. After the feedback phase, data is stored in a central location for review and statistics. Workstations can be installed either at home or at the hospital to support patients, regardless of their location. Two other major initiatives in the field include the research programs set in place by TRIL and CLARITY Centers in Dublin, Ireland. The TRIL Center brings together industry and academia to conduct research studies in older adults and examine how technology can enable health and social care. The CLARITY Center for Sensor Web Technologies is concerned with investigation of the potential of sensor data for playing a key role in the management of personal health. Relevant projects at the TRIL and CLARITY Centers include:

• Development and evaluation of a remote system to assess cognitive function and improve mental alertness among older adults in their homes.

• Building Bridges, a social networking program that allows individuals to communicate with their families and with others on the network without prior experience of computer use.

• Technology to integrate online day reconstruction, psychometric measures, ecological assessments, and biological markers in real-world situations.

• Applications that monitor compliance and provide feedback to patients during the performance of rehabilitation exercise using data gathered via wearable sensors.

Other projects carried out by European groups that are worth mentioning are the TeleKat project and the "Auxilium Vitae Volterra" at Rehabilitation Center-Scuola Superiore Sant'Anna. The TeleKat project (Aalborg University, Aalborg, Denmark) is applying User Driven Innovation to develop wireless tele-homecare technology enabling patients with chronic obstructive pulmonary disease to perform self-monitoring of their status, and to maintain rehabilitation activities in their homes. The Tele-rehabilitation project "Auxilium Vitae Volterra" at Rehabilitation Center-Scuola Superiore Sant'Anna is a cardiac rehabilitation program that leverages the use of a sensor-based system to remotely monitor patients in their home. The system includes a computerized cycle ergometer, a wireless diagnostic 12-lead ECG, a sensor for blood oxygen saturation, a non-invasive blood pressure measurement system, and a high-performance videoconferencing system.

### Assessment of treatment efficacy

A quantitative way of assessing treatment efficacy can be a valuable tool for clinicians in disease management. By knowing what happens between outpatient visits, treatment interventions can be fine-tuned to the needs of individual patients [99]. Another important application would be for use in randomized clinical trials. By gathering accurate and objective measures of symptoms, one could reduce the number of subjects and the duration of treatment required to observe an effect in a trial of a new therapy.

In patients with Parkinson's disease (PD), careful medication titration, based on detailed information about symptom response to medication intake, can significantly improve the patient's quality of life. Medication titration in patients with late stage PD is often challenging as fluctuations in a patient's motor symptom manifest over several hours and hence cannot be observed in a typical outpatient appointment (often lasting no more than 30 min). Patient diaries are unreliable due to perceptual bias and inaccurate reporting about motor status. The above-mentioned issues limit the ability of physicians to optimally adjust medication dosage and to test new compounds for the treatment of PD. The use of a sensor-based system to monitor PD symptoms is a promising approach to improve the clinical management of patients in the late stages of the disease. Major PD symptoms have typical motor characteristics which can be captured using motion sensors such as accelerometers. Manson et al. [100] used a portable tri-axial accelerometer placed on the shoulder to monitor severity of dyskinesia's in PD patients. Dyskinesia's are a side-effect of medication intake and they can cause significant discomfort to patients. Manson and colleagues showed that there is a correlation between accelerometer output and severity of dyskinesia in patients with PD. The ability to estimate the severity of symptoms via processing sensor data recorded during activities of daily living is important for practical applications. Thielgen et al [101] showed that accelerometers can be used to automatically quantify tremor severity scores via 24 hr ambulatory home monitoring in patients with PD. Gait impairments such as shuffling and freezing are characteristics of PD. Paquet et al. [102] have explored the correlation between gait parameters and motor scores in patients with PD. The authors used a biaxial accelerometer mounted on the lower back to measure gait features such as stride frequency, step symmetry and stride regularity. Strong correlation was found between walking regularity and motor scores capturing the severity of PD symptoms. Movement sensors can also be used to automate clinical testing procedures. Salarian et al. [103] and Weiss et al. [104] have proposed instrumented versions of the timed up-and-go test for identifying gait impairments due to PD. They have shown that instrumented tests lead to an improved sensitivity to gait impairments compared to observation methods. Besides, sensor-based methods can also be extended to long term home monitoring. Based on this body of work, an ambulatory gait analysis system, based on wearable accelerometers, for patients with PD has been proposed by Salarian et al. [105].

Intensive long-term rehabilitation post-stroke is an important factor in ensuring motor function recovery. Tracking changes in motor function can be used as a feedback tool for guiding the rehabilitation process. Uswatte et al. [106,107] have shown that accelerometer data can provide objective information about real-world arm activity in stroke survivors. In their study, 169 stroke survivors undergoing constraint-induced movement therapy wore an accelerometer on both wrists for a period of 3 days. The results indicated good patient compliance and showed that by simply taking the ratio of activity recorded on impaired and unimpaired arm using accelerometers, one can gather clinically-relevant information about upper extremity motor status. Prajapati et al. [108] performed a similar study for the lower extremities. The authors used two wireless accelerometers placed on each leg to monitor walking in stroke survivors. Results showed that the system was able to monitor the quantity, symmetry and major biomechanical characteristics of walking. Finally, Patel et al. [109] showed that, using accelerometers placed on the arm, it is possible to derive accurate estimates of upper extremity functional ability. The authors used a small subset of tasks from the Wolf Functional Ability Scale (FAS) to derive estimates of the total FAS score via analysis of the accelerometer data. As the tasks selected from the FAS closely resemble tasks performed during the performance of activities of daily living, such a system could be used for unobtrusively monitoring functional ability in the patients' home environment.

### Early detection of disorders

An area of growing interest in the field of wearable technology is the use of wearable sensors and systems to achieve early detection of changes in patient's status requiring clinical intervention. An example of this type of application of wearable technology is the management of patients with chronic obstructive pulmonary disease. A major goal in the clinical management of patients with chronic obstructive pulmonary disease is to achieve early detection of exacerbation episodes. Exacerbations, commonly defined to be episodes of increased dyspnea, cough, and change in amount and character of sputum, are a prominent part of the natural history of chronic obstructive pulmonary disease, resulting in functional impairments and disability. Early detection and treatment of exacerbations are important goals to prevent worsening of clinical status and the need for emergency room care or hospital admission. Remote monitoring systems can play an important role in early detection of trends in patients' health status that point towards an exacerbation event.

One way to approach the problem of achieving early detection of exacerbation episodes is to detect changes in the level of activity performed by a patient [110,111] and assume that a decrease in activity level would be indicative of the likelihood of a worsening of the clinical status of the individual undergoing monitoring. Atallah et al. [112] have developed an ear worn sensor that can be used to monitor activities and levels of exertion in patients with chronic obstructive pulmonary disease. Using sophisticated machine learning algorithms, the authors were able to identify several different types of physical activities and the intensity of those activities from a single ear worn sensor. Steele et al [113] and Belza et al [114] measured human movement in three dimensions over 3 days and showed that the magnitude of the acceleration vector recorded in patients with chronic obstructive pulmonary disease was correlated with measures of patient's status such as the six-minute walk distance, the FEV1 (Forced Expiratory Volume in 1 sec), the severity of dyspnea, and the Physical Function domain of health-related quality of life scale. Hecht et al [115] presented an algorithm for a minute-by-minute analysis of patients' activity level, based on data recorded using a single unit. The system was tested in 22 patients who were monitored over a period of 14 days. The authors also implemented a simple empirically-developed algorithm to determine if the subject was wearing the device thus providing a handle on compliance. Another interesting observation from the same study was that subjects tended to increase their activity level during the first few days of monitoring. This observation suggests that it is important that monitoring, if performed periodically, be performed over periods of time sufficient to avoid observing transitory effects introduced by the fact that the subject is aware of being monitored.

Combining physiological sensors with activity monitors is a promising way of identifying not only the type of activity performed by subjects undergoing monitoring but also the intensity with which the activity was performed. Furlanetto et al [116] and Patel et al. [117] showed that a multi-sensor system, which measured galvanic skin response, heat flow and skin temperature in addition to motion, provided accurate estimates of energy expenditure. Although not accurate at step counting, the multi-sensor system outperformed the step counters in estimating energy expenditure at slow walking speeds. With the development of wearable sensors and systems [118,119], which can be used for simultaneous monitoring of activities and several physiological parameters such as heart rate, respiration and oxygen saturation, it becomes possible to envision a more comprehensive status monitoring of patients with chronic obstructive pulmonary disease.

Another condition that has been studied extensively in the context of field monitoring is dementia. Dementia refers to a collection of symptoms that describe impairment in cognitive function. More than 30 million people suffer from dementia worldwide and account for approximately $315 billion in medical care costs. Most of these costs are attributed to the use of nursing care facilities. Allowing patients to stay at home longer can lead to significant savings in medical care costs. Remote monitoring can play an important role in the management of patients with dementia. Systems that can assist these patients with remembering daily activities and monitoring daily behavior for early signs of deterioration can allow patients to live independently longer. Such systems range from monitoring activities of daily living to tracking medication compliance to monitoring changes in social behavior. In this context, it is of particular interest to assess the severity of dementia and its changes over time. Haiying et al [120] developed a remote monitoring system for analysis of sleep patterns in patients with early dementia. By performing objective monitoring of quality, quantity and rhythm of sleep the authors aimed to identify the level of cognitive impairment of individuals undergoing monitoring. The monitoring system included passive infrared (PIR) and bed pressure sensors. Preliminary results suggested that the sleep patterns of patients suffering from mild dementia are of lower quality when compared to control subjects. Other efforts to achieve the goal of assessing the progression of dementia have been made by other research groups. Among others, Jimison et al. [121] developed a simple monitoring system based on the modified version of a standard computer game for early detection of dementia. Another important factor in this patient population is that the monitoring system must be totally unobtrusive and if possible collect information in a transparent way without patient intervention due to their cognitive impairment. To achieve this goal, Hayes et al. [122] developed a home monitoring system based on distributed infrared motion sensors and contact sensors. The system was used to assess activity patterns in 14 individuals with mild cognitive impairment. The sensor system was completely unobtrusive. The results of the study showed that daily activity patterns of individuals with cognitive impairments tend to be more variable than healthy controls.

## Conclusions

Whereas the first decade of research in the field of wearable technology was marked by an emphasis on the engineering work needed to develop wearable sensors and systems [123], recent studies have been focused on the application of such technology toward monitoring health and wellness. This consideration was the basis for this review paper. This paper summarized enabling technologies developed over the past decade [6] and put a great deal of emphasis on surveying studies focused on the deployment of wearable sensors and systems in the context of a concrete clinical applications, with main focus on rehabilitation. The interest of researchers and clinicians for pursuing applications of wearable sensors and systems has caused a shift in the field of wearable technology from the development of sensors to the design of systems. Consequently, we have witnessed a great deal of work toward the integration of wearable technologies and communication [16] as well as data analysis technologies so that the goal of remote monitoring individuals in the home and community settings could be achieved. Besides, when monitoring has been performed in the home, researchers and clinicians have integrated ambient sensors in the remote monitoring systems. We have also witnessed a growing interest for the emerging need for establishing a telepresence in the home setting to implement clinical interventions. We envision that home robots will soon be integrated into home monitoring systems to facilitate achieving the goal of establishing a telepresence in the home environment [7,124]. Research toward achieving remote monitoring of older adults and subjects undergoing clinical interventions will soon face the need for establishing business models to cover the costs and identify reimbursement mechanisms for the technology and its management. We envision that addressing costs and reimbursement problems will be essential to assure that wearable sensors and systems deliver on their promise of improving the quality of care provided to older adults and subjects affected by chronic conditions via remote monitoring of wellness and heath in the home and community settings.

## Competing interests

The authors declare that they have no competing interests.

## Authors' contributions

Each author participated in the drafting of the manuscript. All authors approved the final manuscript.
